# Utility analysis and demonstration of real-world clinical texts: A case study on Japanese cancer-related EHRs

**DOI:** 10.1371/journal.pone.0310432

**Published:** 2024-09-11

**Authors:** Shuntaro Yada, Tomohiro Nishiyama, Shoko Wakamiya, Yoshimasa Kawazoe, Shungo Imai, Satoko Hori, Eiji Aramaki

**Affiliations:** 1 Division of Information Science, Graduate School of Science and Technology, Nara Institute of Science and Technology, Nara, Japan; 2 Artificial Intelligence and Digital Twin in Healthcare, Graduate School of Medicine, The University of Tokyo, Tokyo, Japan; 3 Division of Drug Informatics, Faculty of Pharmacy, Keio University, Tokyo, Japan; Auna Ideas, PERU

## Abstract

Real-world data (RWD) in the medical field, such as electronic health records (EHRs) and medication orders, are receiving increasing attention from researchers and practitioners. While structured data have played a vital role thus far, unstructured data represented by text (e.g., discharge summaries) are not effectively utilized because of the difficulty in extracting medical information. We evaluated the information gained by supplementing structured data with clinical concepts extracted from unstructured text by leveraging natural language processing techniques. Using a machine learning-based pretrained named entity recognition tool, we extracted disease and medication names from real discharge summaries in a Japanese hospital and linked them to medical concepts using medical term dictionaries. By comparing the diseases and medications mentioned in the text with medical codes in tabular diagnosis records, we found that: (1) the text data contained richer information on patient symptoms than tabular diagnosis records, whereas the medication-order table stored more injection data than text. In addition, (2) extractable information regarding specific diseases showed surprisingly small intersections among text, diagnosis records, and medication orders. Text data can thus be a useful supplement for RWD mining, which is further demonstrated by (3) our practical application system for drug safety evaluation, which exhaustively visualizes suspicious adverse drug effects caused by the simultaneous use of anticancer drug pairs. We conclude that proper use of textual information extraction can lead to better outcomes in medical RWD mining.

## Introduction

The US FDA defines real-world data (RWD) in the medical field as data related to patient health status or the delivery of healthcare routinely collected from various sources [1]. These sources include social media, wearable/mobile devices, claims or billing activities, disease registries, and electronic health records (EHRs). A considerable part of the RWD data reported in previous studies is structured tabular-order data [2].

Textual notes, which are often recorded in EHRs, are a notable format for RWD. Although the notes contain rich information about patients, to fully utilize EHR data, we must handle natural language texts, i.e., *unstructured* data [3]. Natural language processing (NLP) techniques have gained importance in the medical field for extracting and organizing medically meaningful information from text. An increasing number of medically practical NLP applications (MedNLP) are proposed annually. NLP methods have enabled several clinical applications, including phenotyping [4], incident detection [5], and early diagnosis [6]. While recent NLP has been based on machine learning (ML), ISPOR, which established guidelines for the medical application of ML, also concluded that NLP is useful for cohort selection [7]. Named entity recognition (NER) and relation extraction (RE) underlie these studies. NER identifies medical terms and phrases (named entities; NEs) from raw text, such as disease names [8] and medication names [9]. RE determines the medical relationships between NEs identified by NER. For instance, diseases can be linked to their anatomical locations (e.g., “*cancer* in the *left lung*”) [10], whereas medications are tethered to their dosages (e.g., “take *25 mg* of *metoprolol*”) [11].

Despite the large number of fundamental MedNLP techniques, only a few have validated their practices in real-world medical settings (e.g., in hospitals) [12]. This is partly because the utilization of NLP is not trivial for non-NLP experts. In addition, evaluating clinical MedNLP applications requires collaboration with hospitals, where most NLP researchers do not have prerequisite connections. For instance, the identification of adverse drug effects from EHRs is a popular topic in MedNLP because of its potential for drug safety evaluation. However, it has not yet been well implemented in clinical practice [13].

The present study tackles this situation: to obtain medically meaningful insights using NLP, we evaluate the information gained by supplementing the data of legacy structured tables (TABLE) with clinical concepts extracted from unstructured EHR texts (TEXT). Furthermore, we demonstrate that such gained information can be used in a practical medical application. Using a Japanese hospital database and focusing on two-centric medical information, diseases and medications, we address the following three research questions (RQs) to explore the utility and feasibility of NLP-powered RWD mining.

### RQ1: How different is the retrievable information between TABLE and TEXT?

We investigated the distribution of entities found in TABLE and TEXT to measure the nature of the retrievable information. Specifically, we compared the frequencies of disease and medication names. This analysis revealed the information extracted from TEXT that could effectively supplement the TABLE data.

### RQ2: How much do different sources intersect regarding retrievable disease information?

In contrast to RQ1 depicting the different nature (quality) of TABLE and TEXT, medical data mining practitioners are also concerned about the amount (quantity) of information retrieved from structured and unstructured data. Following prior work [3], we drew Venn diagram intersections for the number of patients with several diseases per data source. This visualization shows how targeted patients can be identified using either TABLE or TEXT. The size of the intersections also suggests the extent to which the combination of TABLE and TEXT can further guarantee this phenotyping or cohort selection process, which can vary among different diseases.

### RQ3: How can both data sources be applied to a practical issue, such as drug safety evaluation?

RQ1 asks what medical information should be extracted from which sources, TABLE and TEXT. RQ2 indicates how many patients with a specific disorder can be identified from a combination of TABLE and TEXT. Based on the RQ 1 and 2 findings, a drug safety evaluation was set as a possible case study to demonstrate a MedNLP application using RWD, combining medication data from TABLE and symptom information (potential adverse reaction) from TEXT. We explored adverse effects potentially caused by certain anticancer drug pairs (or adverse drug-drug interactions [14]) by visualizing the case frequencies in heatmaps. A web-based tool for visualization was developed as a reference.

The results of this study could provide a basis for NLP-based RWD mining and its applications, which would encourage future medical studies and practices.

## Material & methods

### Data sources

The following two resources were obtained from the EHR database of the University of Tokyo Hospital, Japan:

- **TABLE**: diagnosis procedure combinations (**DPC**s) and medication orders (**MO**s)
- **TEXT**: unstructured free text of discharge summaries

Note that all the string entries in the TABLE and the entire text of TEXT are written in Japanese.

This study primarily targeted patients with cancer because cancer is the leading cause of death in Japan. This makes the investigation of our NLP-based RWD mining a priority. For TABLE, we were allowed to use a patient pool of those hospitalized between 2004-01-01 and 2021-12-31 who were between 16 and 100 years of age at admission. In April 2023, cancer patients were identified by the corresponding International Classification of Diseases (ICD) codes (starting with “C”) recorded as the ‘primary disease’ in the pool, resulting in 44,502 patients with 138,656 events; each *event*, the unit of our analysis, denotes one discharge.

**Fig 1:**
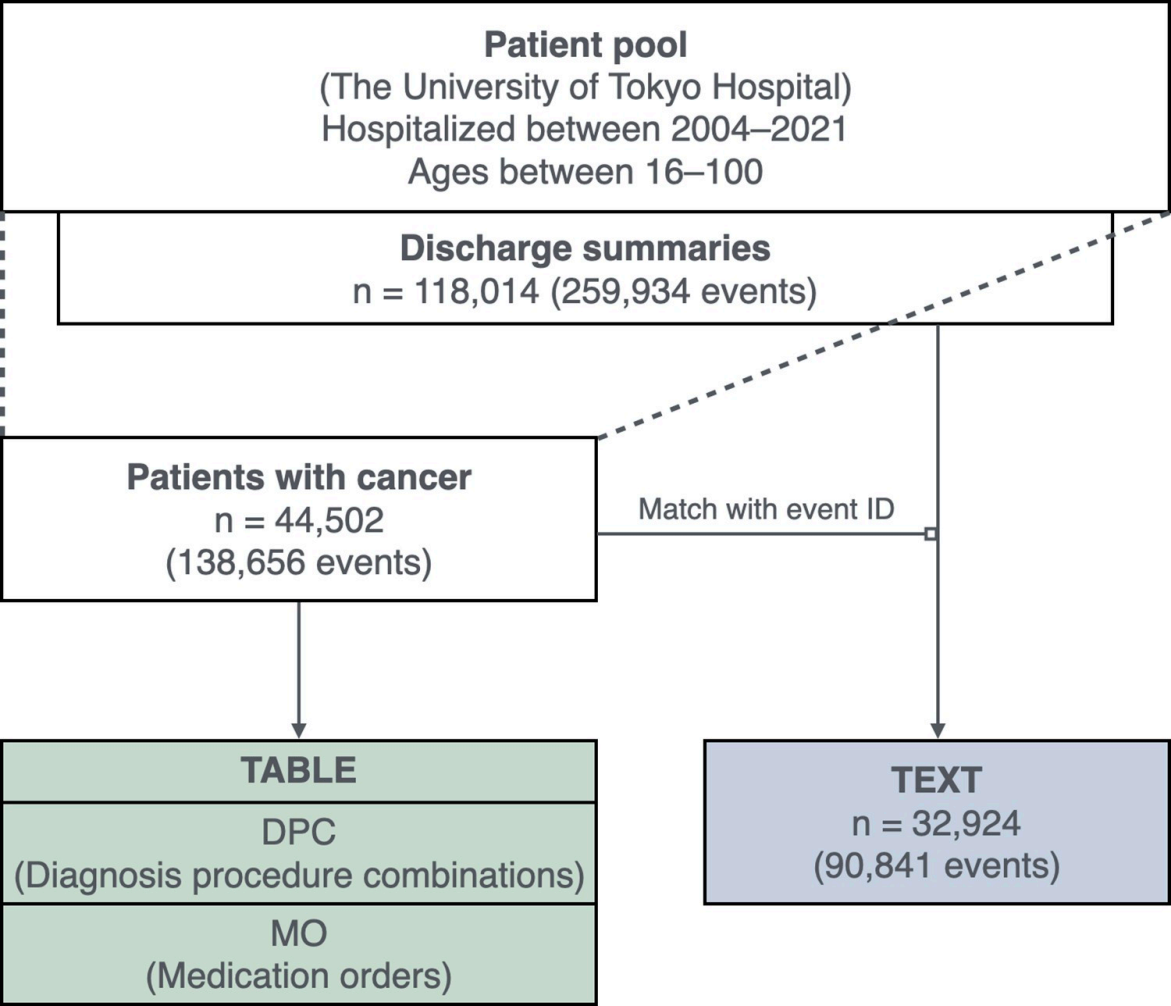
Flow of the data preprocessing. Based on event IDs, we aligned patients and events between the two sources, TABLE and TEXT.

For TEXT, we accessed 259,934 discharge summaries from 118,014 patients obtained from the same patient pool. One discharge summary corresponds to an *event*. Matching the event IDs with those of the TABLE, 90,841 events (discharge summaries) of 32,924 patients were selected; hence, the targeted events were included within those of TABLE, as shown in Fig 1.

**TABLE.** The TABLE consists of **DPC**s and **MO**s; both relational database tables are associated with an anonymized *event* ID column. Owing to the nature of the original database, we can safely regard one DPC as an *event* (i.e., one time of discharge or hospitalization). On average, one patient experienced 3.12 events (std: 4.06). Along with fundamental patient information (e.g., sex, date of hospitalization, department, and the number of times of operations, to name a few), a DPC record consists of two columns of the primary diseases that caused patient hospitalization, and other significant diseases, e.g., two columns of “the disease or symptom that required the (second) most medical resources during hospitalization” and 20 columns of “the disease or symptom observed after the hospitalization or already known before the hospitalization.” These 24 disease-related columns contain the corresponding ICD codes we focused on for the present analysis.

An MO record contains medication product names, their routes of administration (oral or injection), and other metadata (e.g., the amount and start date, to name a few). Each MO record is associated with an event ID; thus, all the medications ordered for an event can be listed. The names and routes of medications were the primary focus of the study. We linked slightly different names of identical medications based on the Japanese medicine dictionary Hyakuyaku [15]. Variations in medication names were linked to pharmaceutical ingredients based on the edit distance (normalized Levenshtein distance), with a score of 70 as the cutoff value.

**TEXT.** Doctors input discharge summaries as dedicated free-text entries in the original EHR system in the hospital. Therefore, TEXT consists of unstructured bare Japanese texts, which means NLP is required to extract the medical information. The methodology is explained below.

### NLP preprocessing on TEXT

**Fig 2:**
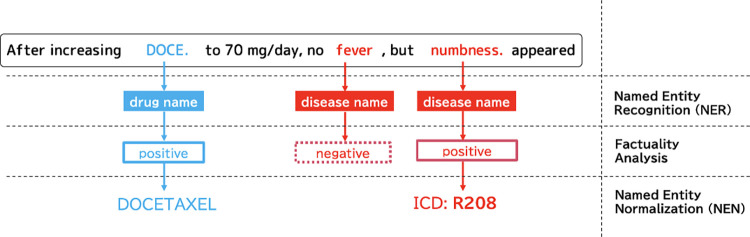
Three-step NLP-based medical information extraction. (1) Named Entity Recognition (NER), (2) Factuality Analysis, and (3) Named Entity Normalization (NEN).

TEXT was processed with NLP-based information extraction to extract information about diseases and medications related to the events (Fig 2). First, we applied an NER tool called MedNER-CR-JA [16], which recognizes more than ten types of medical expressions (*entities*) from Japanese texts, such as disease, anatomical parts, medicine, and tests, following the medical entity scheme proposed by Yada et al. [10]. This analysis focused on *disease* and *medicine (drug)* entities.

Second, we filtered out negated entities of disease and drug, e.g., the “cancer” in ‘*cancer* was not found,’ and the “docetaxel” in ‘the docetaxel has been canceled.’ MedNER-CR-JA can simultaneously perform this negation detection (or *factuality analysis*) while recognizing entities.

Third, we applied normalization to the extracted disease and drug entities, matching diverse medical phrases to the corresponding medical concepts (*named entity normalization; NEN*). We created another Japanese medical-phrase normalization dictionary, ADE-TABLE, by manually augmenting the Japanese disease dictionary Manbyo [17], and the aforementioned Hyakuyaku [15]. Each dictionary entry includes a surface expression, its medical concept, and seven popular adverse drug effects (ADEs) in cancer treatment (see samples in S1 File). For each entity, the corresponding standard term was searched by fuzzy matching to surface expressions based on the edit distance, where a score of 70 in the normalized Levenshtein distance was used as the cutoff value after manual speculation of a few NEN results. This relaxed matching strategy could increase the number of entities for analysis, although it should be noted that some concept-linking errors were introduced.

Fig 2 illustrates this workflow using the example of an EHR sentence. Three entities can be extracted from this sentence using NER. We handle only two types of medical entities: drug names (“DOCE”) and disease names (“fever” and “peripheral neuropathy”). Subsequently, we filtered out the negative entity “fever,” which was not identified to the patient. Finally, disease and drug entities were normalized to their medical concepts: ICD10 codes and pharmaceutical ingredient names. Consequently, two normalized entities are obtained from this sentence: “DOCETAXEL” and “R208.”

### Analysis methods

#### RQ1: Entity frequency comparison between TABLE & TEXT

The diseases and medications mentioned in the TABLE and TEXT were explored based on entity lists per event. For TABLE, disease and medication entities were retrieved from the DPCs and MOs. Disease entities in DPCs correspond to the strings and ICD codes inputted in the 24 disease-related columns; medication entities in MOs are equal to those in the ‘medication name’ column. For TEXT, NLP preprocessing provides disease and medication entity lists per event.

We compared these per-event entity lists from the following perspectives: *entity*, *normalized entity*, and *concept*. Regardless of NEN, the *entity* frequency counts each appearance of the disease or medication phrases. *Normalized entities* are those linked to medical concepts by NEN, which may include normalization errors. The normalized entity frequency excludes entities that are not linked to any concept by NEN. *Concept* frequency counts the medical concepts, which equals the number of unique concept-wise normalized entities. We investigated the common and different parts of the statistics of entity and concept frequencies in total and per event.

Furthermore, we visualized the results using frequency tables of disease and medication concepts to compare content. In addition, for medicine, we provide a 2D scatter plot of medications in terms of frequencies both in TABLE and TEXT, where trends are compared with different routes of administration (i.e., oral and injection).

#### RQ2: Venn diagrams of patient identification

Following Wei et al. [3], we investigated the overlap of retrievable information from different sources of clinical information using Venn diagrams. This roughly replicates the process of phenotyping and cohort selection, identifying patients suffering from specific disorders or showing particular symptoms. We examined the following data sources:

- **TABLE**:
- **DPC**: Count the patients observing the target disease’s ICD codes from all the 24 disease-related columns
- **MO**: Count the patients who received medications that were only or mainly used for the target disease, where such medications were selected based on the ATC codes by one of the authors with pharmaceutical experience (the entire list is available in S2 File).
- **TEXT**: Count patients whose events contain any normalized entities of the target disease concept (ICD).

The eight diseases below were selected for analysis. The corresponding (ranges of) ICD10 codes were manually curated using a Japanese ICD10 code lookup tool developed by the Medical Information System Development Center, Japan. These ICD10 codes and subcategories were used for the patient identification process.

- **Alzheimer’s disease**: G300, 301, 308, 309
- **Breast cancer**: C50
- **Diabetes**: E10-14
- **HIV infection**: B20-24
- **Hypercholesterolemia**: E780
- **Hypertension**: I10-15
- **Parkinson’s disease**: A521, G20, G211, G213, G214, G219
- **Rheumatism**: M123, M080, M05, M06

Although our data sources originated from cancer patients, we included several diseases that would rarely coincide with cancer to (i) ensure that such rare cases appear in expected small numbers, (ii) provide reference points to prior work [3], and (iii) show the utility of this method in locating rare, but potentially important signals.

#### RQ3: ADE candidate exploration

The adverse effects of anticancer drugs serve as a pertinent example of applying NLP to RWD because we target cancer patients. Anticipating the findings of RQ 1, potential adverse effects can be better extracted from TEXT, whereas medication information is more precise in TABLE. Moreover, the results of RQ 2 suggest that an overlapping patient cohort would exist between TABLE and TEXT for this cancer-relevant phenomenon.

We first selected events that ordered anticancer drugs. The number of events that applied each drug pair (*M*) was counted. Among these events, we calculated the number of the events that observed the target symptom, either stomatitis or peripheral neuropathy (*D*). Finally, we draw heat maps of the percentage of *D*/*M* (element-wise divisions of arrays), named *co-frequency ratio heatmaps*. We targeted *stomatitis* and *peripheral neuropathy*, which are frequent adverse drug effects (ADEs) induced by specific anticancer drugs [18, 19]. We investigated the co-occurrences of anticancer drug pairs with each targeted symptom per hospitalization because these drugs are often administered as a combination therapy,.

To demonstrate the utility of this analysis method for medical RWD mining practitioners, we developed a visualization system called **ADE Explorer** to browse the co-frequency ratios of known ADE symptoms and drug pairs per drug category.

#### Ethics statements

All experiments and data collection procedures were approved by the Institutional Review Board of the University of Tokyo Hospital (approval number 2022251NI). The EHR databases were anonymized from the data extraction stage described above; thus, we could not identify any patients throughout the research process. All the experiments described below were performed under the relevant ethical guidelines and regulations.

## Results & discussions

### RQ1: Frequency comparison between TABLE & TEXT

Table 1 shows the results of the entity and concept frequencies of diseases and medications extracted from TABLE and TEXT. Note that the values in “total events that contain entities” are smaller than the total event size mentioned earlier (138,656 for TABLE and 90,841 for TEXT) because some events may not contain any disease or medication information.

**Table 1:** Statistics of TABLE and TEXT.

	Disease (and symptom)		Medication (drug)	
	TABLE (DPC)	TEXT	TABLE (MO)	TEXT
**Total events that contain entities**	133,498	81,690	125,487	72,878
**Total entities**	554,308	3,438,265	10,384,586	1,382,462
**Total unique entities (vocabulary)**	10,195	272,808	8562	72,928
**Total common entities**	3,998		209	
**Total normalized entities**	–	1,873,956	8,614,858	832,524
**Normalized entity rate**	(100.0%)	54.5%	83.0%	60.2%
**Total concepts**	2,883	3,350	1,265	5,412
**Total common concepts**	2,204		525	
**Median entities/event**	3.00	28.00	29.00	11.00
**Mean entities/event**	4.15	42.09	82.75	18.97
**Std entities/event**	1.92	43.06	221.81	23.00
**Median normalized entities/event**	–	16.00	23.00	6.00
**Mean normalized entities/event**	–	22.94	68.65	11.42
**Std normalized entities/event**	–	22.95	182.23	15.32
**Median concepts/event**	1.00	8.00	9.00	4.00
**Mean concepts/event**	2.15	9.91	11.26	5.35
**Std concepts/event**	1.77	7.16	8.45	5.41

“Normalized entity rate” equals “total normalized entities” divided by “total entities,” roughly corresponding to how much the normalization process kept the information. Although more than 80% of the total entities from TABLE were normalized (note that DPC entities are linked to the concepts in advance, resulting in 100% normalization), the entities retrieved from TEXT were more challenging to normalize (e.g., approximately 55–60%). Although we believe that the total number of entities was sufficiently large for the present analysis, this difference encourages the development of a high-performance NEN technique (in Japanese). Certain disease or medication phrases may correspond to multiple medical concepts. These were excluded from the present analysis and left for future research.

We described the disease and medication-specific discussions below.

#### Richer disease information in TEXT and more medication data in TABLE

As shown on the left side of Table 1, the mean of the disease concepts in TEXT was 9.91, which was much greater than that in TABLE (i.e., 2.15). This indicates that TEXT presents richer information than TABLE. TABLE contains, for most events, only the disease names that need to be assigned for insurance claims, which are usually one (i.e., 1.00 in the median concepts/event). In contrast, the TEXT, originating from discharge summaries, contains many patient symptoms as shown in the subsequent analysis.

Conversely, the mean score of the medication concepts in TABLE was 11.26, as shown on the right side of Table 1. This value was more than twice that of TEXT (i.e., 5.35). This is not surprising because TABLE comprises the records of medication orders exhaustively managed by the hospital, including repetitive orders of saline. Alternatively, it has been suggested that discharge summaries (TEXT) tend only to include drugs that concern physicians.

#### The gap in frequent disease entities

Table 2 presents the most frequent disease concepts in TABLE and TEXT to investigate the differences between the two sources. Symptom concepts (ICD10 codes starting with ‘R’), such as ‘fever’ and ‘hypertension,’ appeared only in TEXT. However, the concepts in TABLE were disease names (e.g., cancer-type names such as ‘lung cancer’ and ‘stomach cancer’), which did not often appear in the corresponding TEXT entries. This implies usage segregation in disease/symptom recording in hospitals: TABLE is for primary diseases, and TEXT is for symptoms accompanying hospitalization. Thus, symptom-oriented research is suitable for textual data. Notably, it can uniquely capture subtle diseases and symptoms that would not issue insurance claims (i.e., medical practices), such as ‘fever’ and ‘pain.’ As pointed out in other NLP-based RWD studies [20], this helps automate patient feature abstraction from clinical documents for phenotyping or cohort selection.

**Table 2:** Top 20 disease concepts by normalized-entity frequency in TABLE and TEXT. * indicates the ICD codes defined only in the Japanese version.

	TABLE			TEXT		
	ICD	Description	Freq.	ICD	Description	Freq.
**1**	C220	Liver cell carcinoma	48076	R229	Localized swelling, mass and lump, unspecified	76028
**2**	C56	Malignant neoplasm of ovary	21793	R599	Enlarged lymph nodes, unspecified	53924
**3**	C20	Malignant neoplasm of rectum	21203	D489	Neoplasm of uncertain behavior, unspecified	53529
**4**	C169	Malignant neoplasm of stomach, unspecified	19520	R591	Generalized enlarged lymph nodes	39251
**5**	C61	Malignant neoplasm of prostate	11543	D649	Anemia, unspecified	38267
**6**	C187	Malignant neoplasm of sigmoid colon	9715	R509	Fever, unspecified	38035
**7**	C787	Secondary malignant neoplasm of liver and intrahepatic bile duct	9432	I10	Essential (primary) hypertension	33273
**8**	C341	Malignant neoplasm of upper lobe, bronchus or lung	9262	R17	Unspecified jaundice	31504
**9**	I10	Essential (primary) hypertension	9025	C809	Malignant neoplasm (tumor), primary site unknown	31094
**10**	C549	Malignant neoplasm of corpus uteri, unspecified	8461	E14	Diabetes mellitus, unspecified	30321
**11**	C349	Malignant neoplasm of unspecified part of bronchus or lung	8339	R529	Pain, unspecified	29542
**12**	C539	Malignant neoplasm of cervix uteri, unspecified	8033	C20	Malignant neoplasm of rectum	24454
**13**	C679	Malignant neoplasm of bladder, unspecified	7953	C169	Malignant neoplasm of stomach, unspecified	21439
**14**	C151	Malignant neoplasm of esophagus, chest	7773	R21	Rash and other nonspecific skin eruption	19096
**15**	C343	Malignant neoplasm of lower lobe, bronchus or lung	7406	C787	Secondary malignant neoplasm of liver and intrahepatic bile duct	17662
**16**	K210	Gastro-esophageal reflux disease with esophagitis	6198	C349	Malignant neoplasm of unspecified part of bronchus or lung	17005
**17**	C162	Malignant neoplasm of body of stomach	5936	R609	Edema, unspecified	16937
**18**	C780	Secondary malignant neoplasm of lung	5820	C189	Malignant neoplasm of colon, unspecified	16729
**19**	C159	Malignant neoplasm of esophagus, unspecified	5756	D619	Aplastic anemia, unspecified	16423
**20**	C250	Malignant neoplasm of head of pancreas	5598	J90	Pleural effusion, not elsewhere classified	16278

#### The gap in frequent medication entities

**Fig 3:**
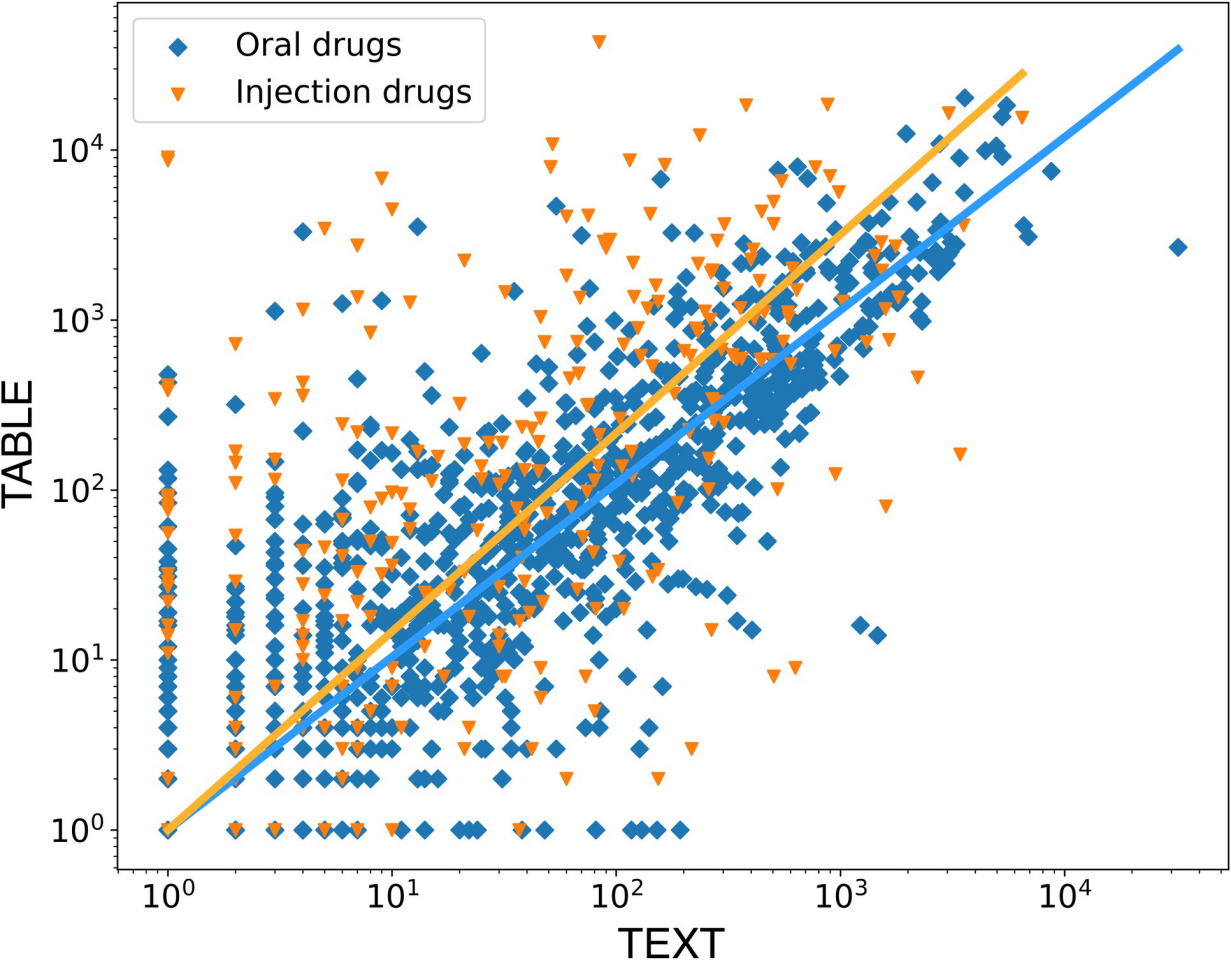
The log-scale plot of the normalized-entity frequency of medications in TEXT and TABLE. The X and Y-axes correspond to the normalized-entity frequencies per medication concept in TEXT and TABLE. The blue rhombuses indicate the count of injection drugs, and the orange triangles indicate that of oral drugs. The regression lines are drawn in the logarithmic scale under the constraint that they pass through the origin.

As shown in Fig 3, the medication entities appear more frequently in TABLE than in TEXT. As mentioned, almost all the administered drugs were included in the medication orders (TABLE), whereas only a handful were described in the note sections (TEXT). Analysis by route of administration showed that oral and injectable drugs produced different plots. Plotting the linear regressions in a log scale with the constraint that they pass through the origin, the coefficients of determination (R2 values) are 1.79 (oral drugs) and 0.99 (injection drugs), respectively. From this, we observed two points. First, the frequency agreement between TEXT and TABLE is higher for oral drugs (blue rhombuses) than for injection drugs (orange triangles). Second, injection drugs appear more frequently in TABLE because many injection drugs are used as ancillary drugs, such as infusion fluids and saline, and these drugs do not typically need to be recorded in TEXT. For reference, the frequency table of medication concepts (a counterpart to Table 2) is attached in the S1 Table.

### RQ2: Venn diagrams

**Fig 4:**
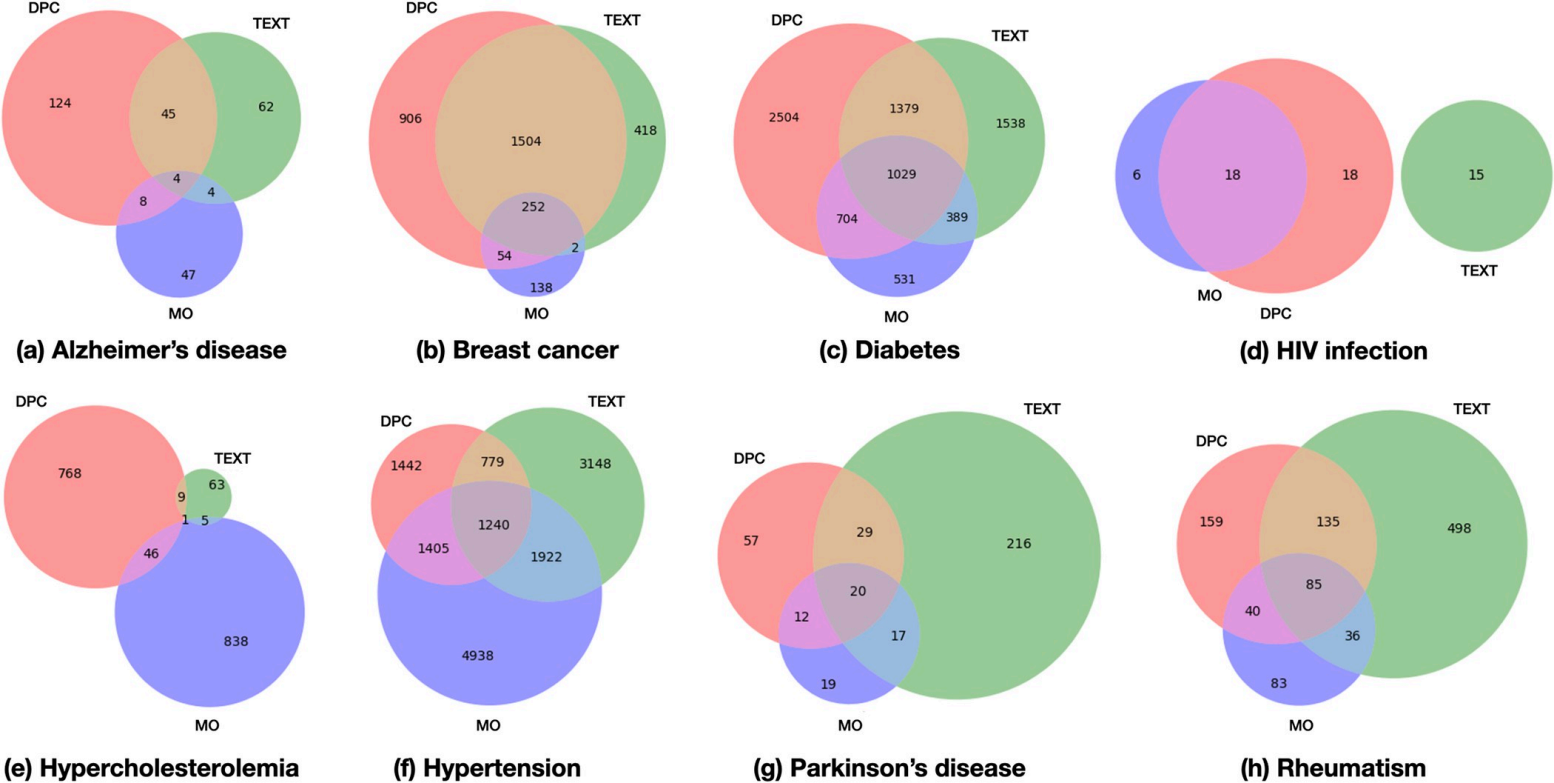
Venn diagrams of the event overlap among three different sources. DPCs and medication orders (MOs) in TABLE, and TEXT.

Fig 4 shows Venn diagrams of the patient intersections. Each number indicates the number of patients from the corresponding data source. Relatively small intersections were observed among the different data sources for all target diseases. This indicates that substantial amounts of disease-related information were dispersed in the different sources. Combining all the sources is recommended for practical RWD mining. All of our sources were derived from patients with cancer. Therefore, the intersection of breast cancer was expectedly large (Fig 4B). In addition, as expected, the diseases that rarely coincide with cancer, such as Alzheimer’s (Fig 4A), HIV infection (Fig 4D), and Parkinson’s (Fig 4G), show small intersections, unlike in Wei et al. [3], who used whole EHR data that was not limited to patients with cancer.

### RQ3: ADE candidate exploration

**Fig 5:**
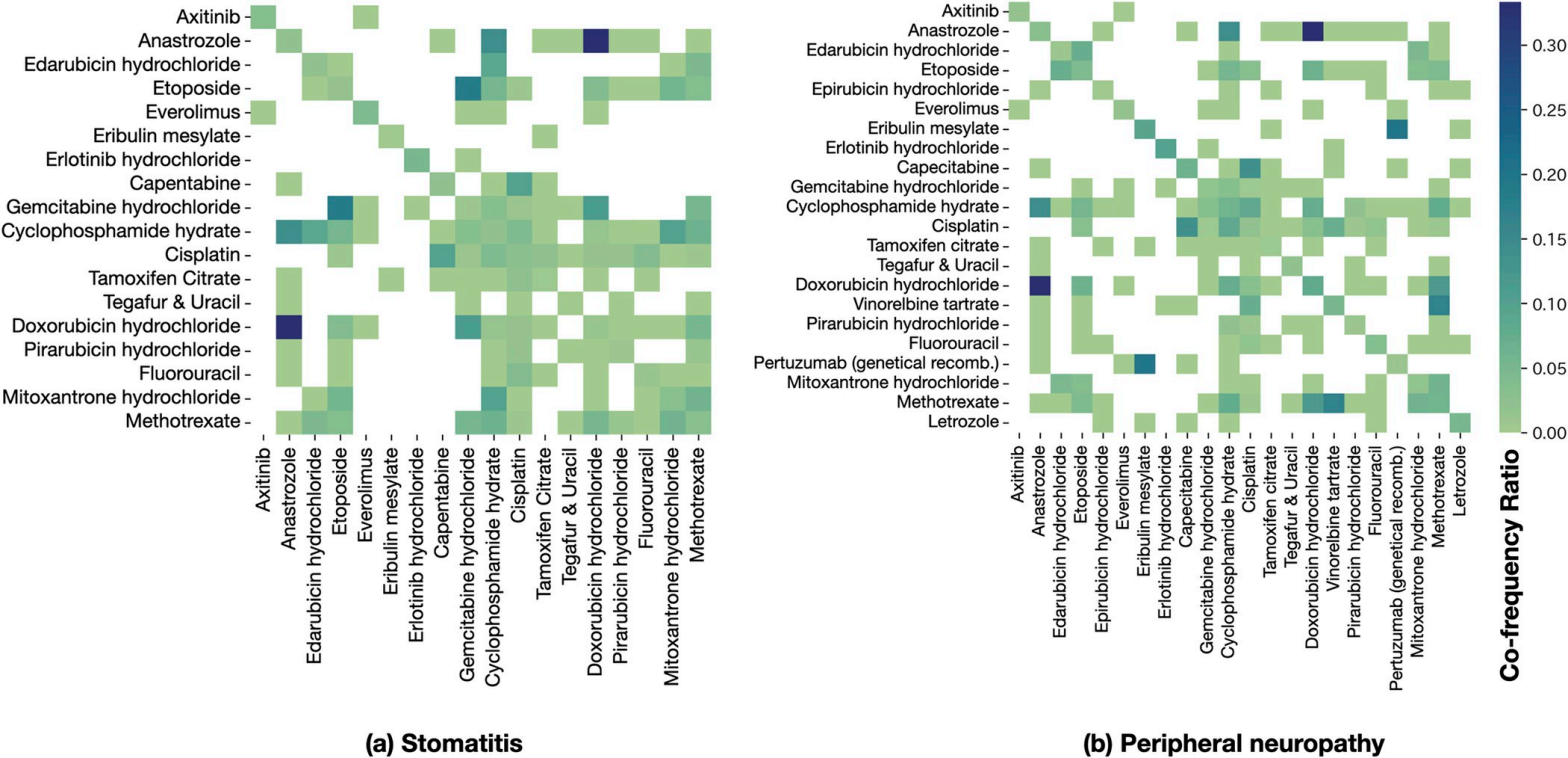
Co-frequency ratio heatmap of anti-cancer drugs and two target symptoms: (a) stomatitis and (b) peripheral neuropathy. The color bar represents the co-frequency ratios of drug combinations (darker is higher).

Fig 5 shows co-frequency ratio heatmaps for stomatitis and peripheral neuropathy. These heat maps support the potential ADEs observed in hospitals. For instance, Fluorouracil and Cisplatin have been reported to cause stomatitis [21, 22], as shown in Fig 5.

Some high-value cells were obtained from a small number of those in the drug-pair frequency matrices (*M*). For instance, only three events were observed for anastrozole and doxorubicin, resulting in a co-frequency ratio of 30%. Rather than this simple division, a finer-grained analysis could be achieved by calculating the odds ratio or setting a minimum cutoff value for the events, which is left for future work.

**Fig 6:**
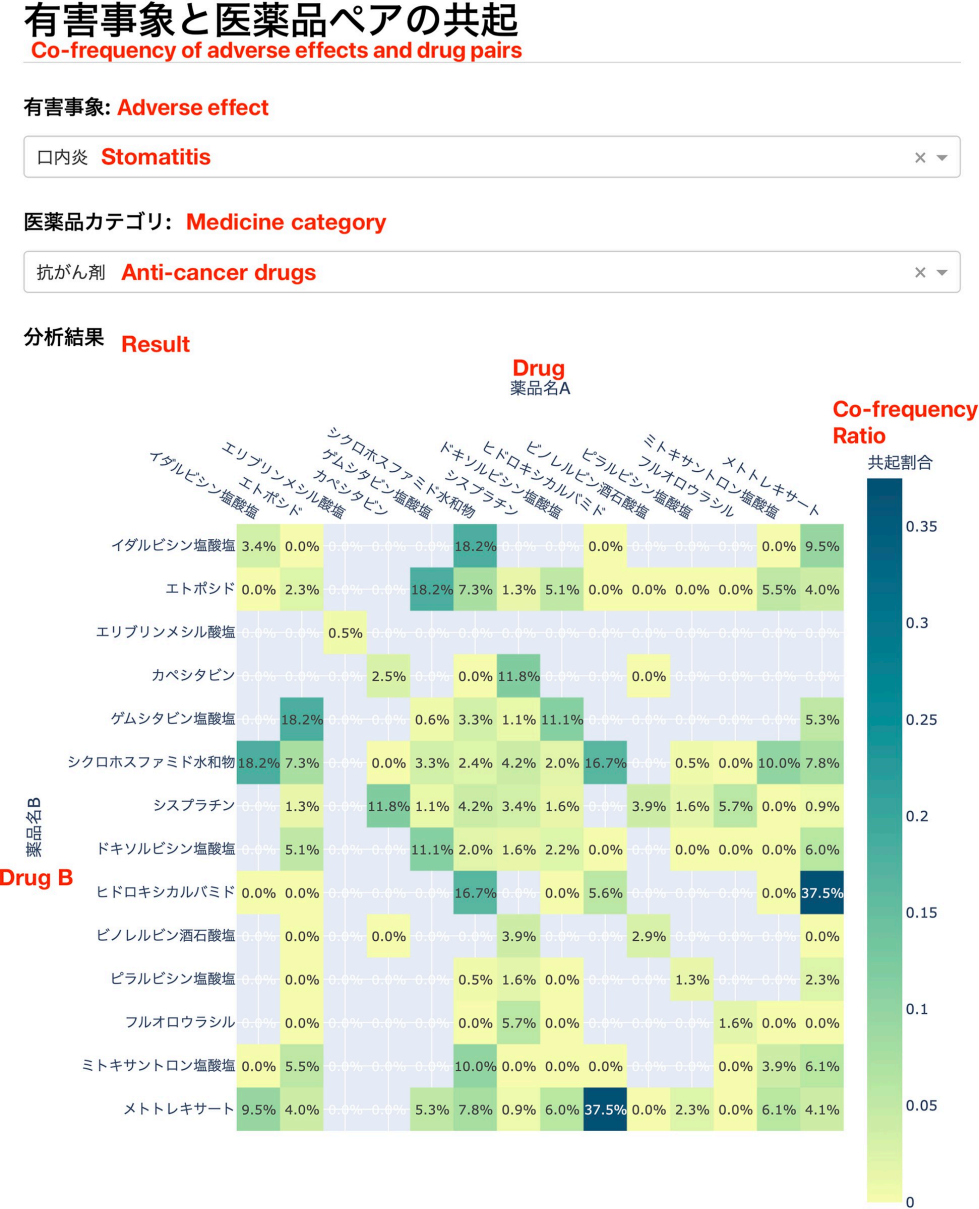
The user interface of ADE Explorer. Users can select a combination of adverse effects (the first selection box) and drug categories (the second selection box). The resulting co-frequency ratio heatmap is shown at the bottom of the screen.

Fig 6 shows the user interface (in Japanese) of the proposed system, ADE Explorer. Other than anticancer drugs, the system allows users to select 200 other drug types, such as tranquilizers, Chinese medicines, and vaccines; more than 30 symptoms are also available to choose from. For demonstration purposes, this system can be accessed at https://aoi.naist.jp/crest-analysis.

## Conclusions

We investigated the differences in patient information extracted from two sources of hospital databases: structured tabular data (TABLE) and unstructured textual data (TEXT), the latter of which was structured using natural language processing (NLP). We observed that more-or-less only the primary diseases are listed in TABLE, whereas TEXT contains a variety of symptoms during patient hospitalization. Regarding medications, TABLE records more injections than oral ones, compared to TEXT. Therefore, these sources cover different ranges of medical information, and RWD mining can be enriched by combining the text. In addition, we demonstrated that textual data-combined RWD mining can be applied to practical medical tasks such as drug safety evaluation. A heat map visualization tool for potential adverse effects caused by anticancer drugs was developed as a reference, which we plan to implement to monitor suspicious adverse effects at the source hospital.

This study has limitations in terms of our material and methodology. Our material covers only patients with cancer from one hospital in Japan, although using an actual database seems valuable. Our methods depend on existing Japanese NLP tools and dictionaries, assuming such tools and resources are mature for the target languages. Applying our approach to low-resource languages could be non-trivial. However, the framework can also be applied to other diseases, hospitals, and languages (including low-resource ones). Replication of our approach in English-spoken hospitals would yield decent quality results because English is the dominant language in medical NLP tools and resources at the present age [12]. Future work could evaluate our approach using publicly available clinical datasets in English, such as MIMIC [23, 24]. We plan to expand our analysis to include a wider range of sites. Finally, our study encourages using NLP-based RWD mining in under-represented languages in medical NLP research.

## Supporting information

S1 File Sample 8 rows of ADE-TABLE. Surface expressions derived from private in-hospital texts in Japanese (TEXT). For readability, we have used their English translations. These surface expressions, which are often symptom-like phrases, are categorized as parent diseases and labeled with corresponding ICD codes. We further label each surface expression with relevance to frequent adverse effects of anti-cancer drugs, such as stomatitis, peripheral neuropathy, and hand-foot syndrome (e.g., <stomatitis> and <peripheral neuropathy>). The original table has eight relevant labels containing binary values (1 if relevant and 0 otherwise). The samples were randomly selected from entries relevant to stomatitis or peripheral neuropathy.
(XLSX)

S2 File Lists of drug names used in the Venn diagram visualization (RQ2) for each target disease. (XLSX)

S1 Table Top 20 medicine concepts by normalized-entity frequency in TABLE and TEXT. These concepts include several pharmaceutical ingredients.
(XLSX)
