# Correction: Utility analysis and demonstration of real-world clinical texts: A case study on Japanese cancer-related EHRs

**DOI:** 10.1371/journal.pone.0320522

**Published:** 2025-03-05

**Authors:** Shuntaro Yada, Tomohiro Nishiyama, Shoko Wakamiya, Yoshimasa Kawazoe, Shungo Imai, Satoko Hori, Eiji Aramaki

The first author, Shuntaro Yada, is incorrectly noted as the corresponding author. The correct corresponding author is Eiji Aramaki. Eiji Aramaki’s email address is: aramaki@is.naist.jp.
